# The causal relationship between neurocysticercosis infection and the development of epilepsy - a systematic review

**DOI:** 10.1186/s40249-017-0245-y

**Published:** 2017-04-05

**Authors:** Lucy B. Gripper, Susan C. Welburn

**Affiliations:** Division of Infection and Pathway Medicine, Edinburgh Infectious Diseases, Edinburgh Medical School: Biomedical Sciences, College of Medicine and Veterinary Medicine, The University of Edinburgh, Chancellor’s Building, 49 Little France Crescent, Edinburgh, EH16 4SB Scotland UK

## Abstract

**Background:**

Neurocysticercosis (NCC) is a parasitic infection of the human central nervous system, the most common form of which involves infection of the brain parenchyma with the larval form of the *Taenia solium* tapeworm. A causal relationship between such an NCC infection and the development of epilepsy in infected individuals is acknowledged, in part supported by high levels of comorbidity in endemic countries worldwide.

**Methods:**

This study undertook a systematic review and critical analysis of the NCC-epilepsy relationship with the primary objective of quantifying the risk of developing epilepsy following NCC infection. A secondary aim was to analyse the proportions of NCC-associated epilepsy within different populations. Significant emphasis was placed on the importance of neuroimaging (CT or MRI) availability and use of clear guidelines for epilepsy diagnosis, in order to avoid overestimations of prevalence rates of either condition; a limitation identified in several previous studies.

**Results:**

A common odds ratio of 2.76 was identified from meta-analysis of case-control studies, indicating that an individual infected with NCC has almost a three times higher risk of developing epilepsy than an uninfected individual. Furthermore, meta-analysis of studies identified a common proportion of 31.54% of epilepsy cases associated with NCC infection which suggests that amongst epileptic populations in at risk countries, approximately one-third may be associated with NCC infection.

**Conclusion:**

A significant finding was the lack of good clinical data to enable accurate determination of a causal relationship. Even studies that were included had noticeable limitations, including a general lack of consistency in diagnostics, and lack of accurate epidemiological data. This review highlights a need for consistency in research in this field. In the absence of reliable estimates of its global burden, NCC will remain of low priority in the eyes of funding agencies - a truly neglected disease.

**Electronic supplementary material:**

The online version of this article (doi:10.1186/s40249-017-0245-y) contains supplementary material, which is available to authorized users.

## Multilingual abstracts

Please see Additional file 1 for translations of the abstract into the six official working languages of the United Nations.

## Background

Neurocysticercosis (NCC) is the most common parasitic disease of the human central nervous system (CNS) [1]. NCC results from ingestion of the eggs of the flatworm *Taenia solium* (commonly referred to as the ‘pork tapeworm’) [2]; the oncospheres hatch in the intestine, penetrate the intestinal wall and disseminate to several body tissues, showing strong tropism to the CNS [3]. Once established the living parasite may be referred to as a ‘cysticercus’ or ‘viable cyst.’ The cysticercus may remain in such a state indefinitely, before an environmental trigger, such as the overcoming of cyst evasion mechanisms by the host immune system, initiates a degenerative transitionary phase. Gradual calcification of the cyst then occurs, until cyst evolution is terminated, whereby it becomes a non-viable, fully calcified nodule [4]. Clinical presentation of these infections are highly pleomorphic, due to the extensive variations in number, location and evolutionary stage of the parasites, with a clinical spectrum of severity that ranges from no discernible symptoms, to rapid increases in intracranial pressure, occasionally leading to death [5]. The absence of a consistent syndrome is one of many factors that hinders making an accurate NCC diagnosis.

NCC has a diverse array of clinical manifestations, depending on a complex range of interconnecting factors, including the number and size of the cysticerci present, their stage of development, and localisation within the brain; this contributes to significant difficulties in accurate diagnosis and staging of the disease [6]. The parasite load, dependent on both the size and number of cysticerci, is an important determinant of symptomatology, with high loads associated with increased risk of obstruction and corresponding rises in intra-cranial pressure (ICP), as well as induction of significant inflammatory responses [5]. In very severe cases, involving numerous cysts with associated inflammation, an encephalitic state can occur with diffuse cerebral oedema; such cases have a very poor prognosis [7].

Despite being considered as the most prevalent neuroparasitic disease worldwide [8], NCC remains on the World Health Organization (WHO) list of neglected tropical diseases, endemic in many developing countries in poverty-stricken areas of the world such as Sub-Saharan Africa and Latin America [8].

Transmission of NCC involves the contamination of food or water with the eggs of *T. solium*, excreted in the faeces of a human tapeworm carrier, meaning that NCC propagation is enhanced by the inadequacy of both sanitation and access to clean drinking water, commonly experienced in developing countries. Pigs are the only other established intermediate host of *T. solium* and, like humans, may develop cysticercosis infection from the ingestion of food or water contaminated with human faeces [4]; in many developing countries it is common practice to allow pigs to roam freely, exacerbating transmission by enabling the frequent ingestion of human faecal matter. The tapeworm cannot reproduce without a definitive human host, and pigs are unable to directly transmit NCC, however ingestion of undercooked pork contaminated with cysticerci can lead to the adult tapeworm infection, or taeniasis, in humans, completing the lifecycle and increasing parasite burden [1].

Despite efforts by organizations such as WHO to increase public awareness of NCC and improved strategies for the control of *T. solium*-related disease, reducing the disease burden is impeded by a number of factors. The biology of the tapeworm itself makes effective control very difficult, as a single adult can produce a minimum of 100 000 eggs in a day [9], which can become distributed in the environment within a large radius of the primary infection [9]; this high biotic potential is a significant factor in the epidemiological stability of the taeniasis-cysticercosis complex [8]. Importantly, strongly established surveillance programs are also lacking in many endemic areas, leading to a limited amount of accurate epidemiological data that is crucial in the assessment of disease transmission and distribution [10]; a greater quantity of such data is key for the development of future control and eradication strategies.

### The neurocysticercosis-epilepsy relationship

Clinical presentation of NCC acknowledges seizures as the most common manifestation of the parenchymal form of the disease, occurring in up to 90% of symptomatic patients [11]. In many cases, the presence of seizures has been interpreted as being synonymous with epilepsy, especially in individuals where seizures are recurrent. It is not uncommon for seizures to be attributed to epilepsy and for NCC to be proposed as ‘a leading cause of epilepsy in the developing world’ [12].

The clear distinctions between epilepsy and seizures have often been overlooked [13]. The International League Against Epilepsy (ILAE) defines epilepsy as ‘two or more unprovoked seizures occurring at least 24 h apart’ [14]. While this is a seemingly clear definition, it is difficult to apply to NCC cases, mainly due to the significance placed on the ‘unprovoked’ nature of seizures. Individuals infected with NCC may remain asymptomatic for prolonged periods, a phenomenon thought to arise from a complex immune evasion response initiated by viable cysts; this allows them to remain undetected in the body for an indefinite timespan [7]. Development of symptoms, including seizures, are often associated with the overcoming of such mechanisms by the immune response, leading to an acute inflammatory reaction [15]; seizures presenting in this context could therefore be considered as having direct provocation. This has led to a spectrum of interpretations as to what constitutes epilepsy as opposed to provoked acute symptomatic seizures, potentially influenced by whether an individual cyst is in the viable, degenerative or calcified state. Inconsistency of such definitions between studies is a major contributor to the difficulties in forming conclusions regarding the causal nature of NCC-associated seizures or epilepsy.

While it is not disputed that there are high rates of NCC-epilepsy comorbidity in certain developing countries [16], determining the existence, or non-existence, of a causal relationship between epilepsy and NCC is far more complex. Considering the nature of the interactions that exist between these two conditions, a number of hypotheses can be considered. Firstly, that NCC is a direct cause of epilepsy. This theory would need to consider the dispute over whether direct provocation by inflammation of, and structural damage to, the brain parenchyma would actually constitute epilepsy versus acute symptomatic seizures.

Secondly, that NCC is a component of the epileptogenic pathway, contributing to the development of the disease, but not acting directly as a causative factor [17]. NCC could be considered an ‘initial precipitating injury’ (IPI) for epilepsy, where NCC infection acts as a trigger for future spontaneous recurrent seizures and the development of an epileptogenic profile, but is not directly responsible for these subsequent individual seizure events [18].

Thirdly that there is an indirect link between epilepsy and NCC due to an independent factor, causing a misleading association between the two conditions. Some studies have shown high rates of familial aggregation of NCC, indicating the presence of a genetic predisposition to the disease that may also be linked to increased risk of epilepsy [19].

Finally, that the high rates of comorbidity of NCC and epilepsy are coincidental due to high concurrent prevalence rates of the two conditions, which occur independently from one another, in certain countries [20, 21].

The aim of this study was to undertake a systematic review and critical analysis of the available literature on the NCC-epilepsy relationship, with the primary objective of determining the risk of developing epilepsy following initial infection with NCC. A secondary objective was to assess the relative proportions of NCC-associated epilepsy in different populations. Analysis of the literature in this area also allowed for consideration of the evidence for and against the four possible hypotheses defining this relationship, to better clarify the mechanisms determining comorbidity.

## Methodology

### Literature search

A systematic search of the literature was conducted using a three-phase approach to efficiently restrict studies for analysis to those containing relevant, high quality data, from a large initial volume of identified studies following the PRISMA guidelines [22]. Phase 1 comprised an initial search for studies documenting the relationship between NCC and epilepsy. Two databases, MEDLINE and Embase, were searched using the Ovid search platform. The following keywords were used in a comprehensive literature search: ‘epilepsy’, ‘seizures’, *‘Taenia solium’,* ‘cysticercus’, ‘cysticercosis’, ‘neurocysticercosis’ and ‘taeniasis’. Exclusion criteria were intended to limit studies to those that included original, relevant data specifically regarding the prevalence or incidence of NCC and epilepsy in defined populations. The following categories of study were excluded: those lacking original data, for example review papers and editorials; those reporting animal data in isolation, with no human subjects; single case reports or studies with very small population sizes (fewer than 20 participants); those containing a lack of appropriate frequency data regarding the association between NCC and epilepsy and those reporting on agents other than *T. solium*. The majority of studies fulfilling these criteria were conducted in the 1990s or later. A screen of a random sample of papers published before 1990 was conducted and yielded no studies for inclusion as they did not meet the criteria.

### Phase 2: full text review

For inclusion for full text review, studies required to have access to neuroimaging equipment, whether CT or MRI, for the visual detection of cysts, to aid accurate diagnosis of NCC. Autopsy studies, where cysts could still be directly visualized and biopsied, were also included. Commonly, immune-serological tests are used in isolation for the clinical diagnosis of NCC, as these are more practical in resource-poor developing countries that are endemic for the disease [22], yet there are a number of issues associated with such methods. The enzyme-linked immune-electro-transfer blot (EITB) assay, which uses targeted antigens to detect serum *T. solium* antibodies, is one of the most widely used assays for NCC diagnosis [2]; however, in endemic countries, where environmental exposure to NCC is high, a significant proportion of individuals from the general population will test positive for antibodies despite not having active NCC infection [23–25]. This can lead to overestimation of NCC prevalence within study populations. More recently, antigen-detecting ELISA assays have been suggested as an improvement over the EITB, as the detection of *T. solium* antigens instead of antibodies indicates the presence of an established NCC infection, as opposed to exposure only [26]. Serological results are useful as a component of a diagnostic toolbox, but only serve to strengthen a suspected diagnosis, interpreted in a clinical context. Combining these results with neuroimaging data was expected to provide the most reliable estimates for NCC prevalence.

Another important inclusion criterion was that methods of epilepsy diagnosis had to meet a certain standard [14, 27]. Given the confusion regarding classification of seizures, and distinction between seizures and epilepsy, studies needed to provide clear definitions of seizure activity in their subjects. Ideally symptoms would be classified according to internationally accepted guidelines, such as those provided by the International League Against Epilepsy [28] (http://www.ilae.org), however as long as general classifications were clearly defined this was not an absolute requirement. Diagnoses could not be based solely on patient history without assessment and confirmation by a qualified clinician, as patient experiences in isolation could introduce an element of recall bias into the determination of proportions of people with epilepsy (PWE).

### Phase 3: data extraction

Prior to data extraction, study characteristics of included papers for review and statistical analysis were assessed and recorded in a Microsoft Excel spreadsheet. Aspects of the studies considered included: year of publication; country in which the study was conducted; the study objective(s); study design e.g., cross-sectional; methods for determining the study population e.g., selection of cases versus controls in relevant studies; size and characteristics of the study population; methods of epilepsy diagnoses and methods used to make NCC diagnoses.

Studies were ordered according to design with case-control studies separated from cohort and cross-sectional studies, since these designs yielded different data types that were used separately in statistical analyses. Data concerning the frequency of NCC and epilepsy cases respectively, within different populations, was extracted and entered into standardized tables, adapted to each study design.

### Statistical analysis

Meta-analysis of case-control data was used to estimate the risk of developing epilepsy following exposure to NCC infection. MedCalc statistical software was used to calculate odds ratios (*OR*s) for each set of study data, with 95% confidence intervals (*CI*s) and *P* values to test for statistical significance. The degree of heterogeneity between studies was measured using the I^2^ index, which assesses the total variability of results due to deviations between studies [29]. A second meta-analysis was carried out on prevalence data from cohort and cross-sectional studies to assess the proportions of NCC-associated epilepsy within different populations. As before, proportions were calculated using MedCalc software, allowing for determination of 95% *CI*s and a measure of heterogeneity.

## Results

**Fig. 1:**
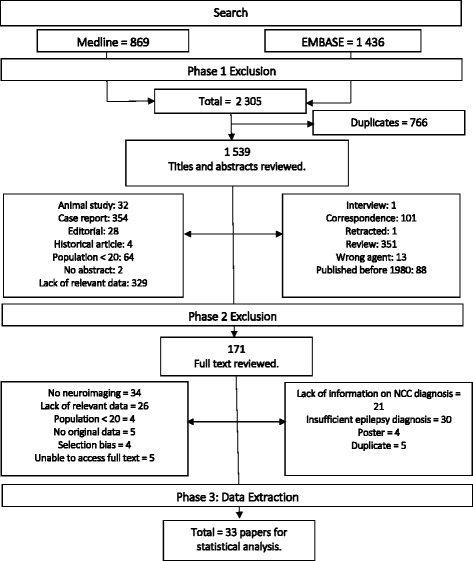
Flowchart showing literature search results over the different exclusion phases

Initial searches of MEDLINE and Embase produced 869 and 1 436 papers respectively. The results of the literature search and subsequent inclusions are detailed in Fig. 1. A reference management application, Mendeley, was used to organise papers, and identified 766 duplications. This left a total of 1 539 papers to be included in the first round of abstract screening.

Papers that lacked original data, such as reviews and editorials were excluded. Single case reports, or studies reporting on very few participants that were structured as case reports, as opposed to considering the population as a whole were also excluded. Other studies were excluded under the ‘lack of relevant data’ criterion including those where NCC or epilepsy featured briefly as a component of a much wider study, with few details regarding their specific diagnosis or assessment. If it was uncertain from the abstract alone whether or not data would be relevant to this review, then it was included for phase 2 evaluation if it met with all further inclusion criteria. In total, 1 368 papers were excluded in phase 1, leaving 171 suitable papers for full text review.

All papers in phase 2 were read and critically reviewed. The greatest proportion of papers excluded in phase 2, were excluded due to a lack of neuroimaging or autopsy for the diagnosis of NCC. Many studies either were conducted in locations that were too geographically remote to practically transport all participants to facilities where CT or MRI were available, or in areas where this equipment was not available even in such facilities.

Thirty papers were excluded due to the way in which the diagnosis of epilepsy was ascertained, mainly due to lack of distinction made between different seizure types, with no defined guidelines concerning classification. A number of papers also only investigated participants who had suffered from a single seizure, with no lifetime recurrence. In a few cases, papers were excluded because participants were classified as epileptic based only on self-reported questionnaire data, with no confirmation from a clinician. 138 papers in total were excluded in phase 2, leaving 33 studies for final inclusion in data extraction and meta-analysis.

The main characteristics of case-control studies that were included are summarized in Additional file 2, and for cross-sectional and cohort studies in Additional file 3. Of 13 included case-control studies, 6 were cross-sectional in origin, and used screening questionnaires to identify PWE within whole communities. The remaining studies generally identified PWE from outpatients in hospitals, or from those attending epilepsy clinics within the study area. The majority of individuals within the control groups was randomly identified from the general population, or was non-epileptic family members of individuals in the case groups; the latter has an advantage in that it allows for a certain degree of genetic matching, whereby the potential for familial aggregation of NCC or epilepsy susceptibility is partially controlled for. When controls were taken from the general population, most studies attempted to match cases in terms of age, sex and socioeconomic background, on a 1:1 basis, however this was often not achieved due to many refusals to participate. In two studies, archived CT data was used as a control group, as opposed to living participants; this was justified due to ethical reasons surrounding the unnecessary risk imposed on healthy individuals in undergoing CT scanning that was not for their own benefit.

A total of 13 studies used in the proportion-based meta-analysis were cross-sectional in design, and a further 13 were cohort-based. All cross-sectional studies identified PWE using screening questionnaires, administered either to whole communities, if small enough, or to random samples of communities if the total population was too large to be included in its entirety. 12 out of 13 cohort studies were hospital-based, 4 of which reported on paediatric populations alone.

Very few studies explicitly stated exact values for the number of individuals concluded to have NCC and/or epilepsy. Regarding NCC, a noticeable amount of studies reported serological and neuroimaging results independently with no comment on how certain a diagnosis these results would constitute. All studies where overall diagnoses were considered used the Del Brutto diagnostic criteria [30] to define NCC cases as either probable or definitive (The Del Brutto criteria include: 1) absolute-histologic demonstration of the parasite from biopsy of a brain or spinal cord lesion, cystic lesions showing the scolex on CT or MRI, and direct visualization of subretinal parasites by funduscopic examination; 2) major-lesions highly suggestive of neurocysticercosis on neuroimaging studies, positive serum enzyme-linked immuneelectrotransfer blot for the detection of anticysticercal antibodies, resolution of intracranial cystic lesions after therapy with albendazole or praziquantel, and spontaneous resolution of small single enhancing lesions; 3) minor-lesions compatible with neurocysticercosis on neuroimaging studies, clinical manifestations suggestive of neurocysticercosis, positive CSF enzyme-linked immunosorbent assay for detection of anticysticercal antibodies or cysticercal antigens, and cysticercosis outside the CNS; and 4) epidemiologic-evidence of a household contact with *Taenia solium* infection, individuals coming from or living in an area where cysticercosis is endemic, and history of frequent travel to disease-endemic areas) [30].

A clear total for individuals diagnosed with epilepsy was also rarely stated, and had to be inferred. Due to these factors, a set of guidelines had to be developed in order to extract data regarding NCC and epilepsy frequency in a consistent manner.

For determination of epilepsy frequency, ideally ILAE guidelines would be stated and used to categorize seizure types, of which epileptic seizures would be one. Where possible, only epilepsy cases defined as active were included. In the absence of such defined data sets, individuals in which seizures were described as recurrent and occurring over a period of more than 24 h were included in the epilepsy totals.

When deciding which cases to include in the totals for NCC infection, the Del Brutto criteria [30] were the preferable referral point, as they were the most consistent set of guidelines used between studies. Where these criteria were used, both definitive and probable NCC cases were included in the total. Where these criteria were not used, NCC cases were defined through the number of cases in which CT or MRI images indicated the presence of NCC, whether lesions were compatible with, suggestive of, or definitive of, NCC. Serological results in isolation were not considered when determining NCC frequency, although they could be included when used in conjunction with the Del Brutto criteria [30].

### Risk of developing epilepsy following NCC infection

Case-control data were used to calculate *OR*s for each study, with associated 95% *CI*s and *p* values. Secka et al. [31] was excluded despite meeting all inclusion criteria, as no individuals from either the case or control group met the criteria for NCC diagnosis; EITB was negative for all participants, and although 11 individuals tested positive on Ag-ELISA, no lesions suggestive of NCC were detected on CT scans. Although these data are useful in suggesting that NCC is not a cause of epilepsy in the Gambia, where the study was conducted, this appears to be due to the fact that NCC is very uncommon in this area, and does not support the more relevant hypothesis that NCC, when present, does not cause epilepsy. The lack of an exposure group does not allow for the calculation of an associated *OR*.

**Fig. 2:**
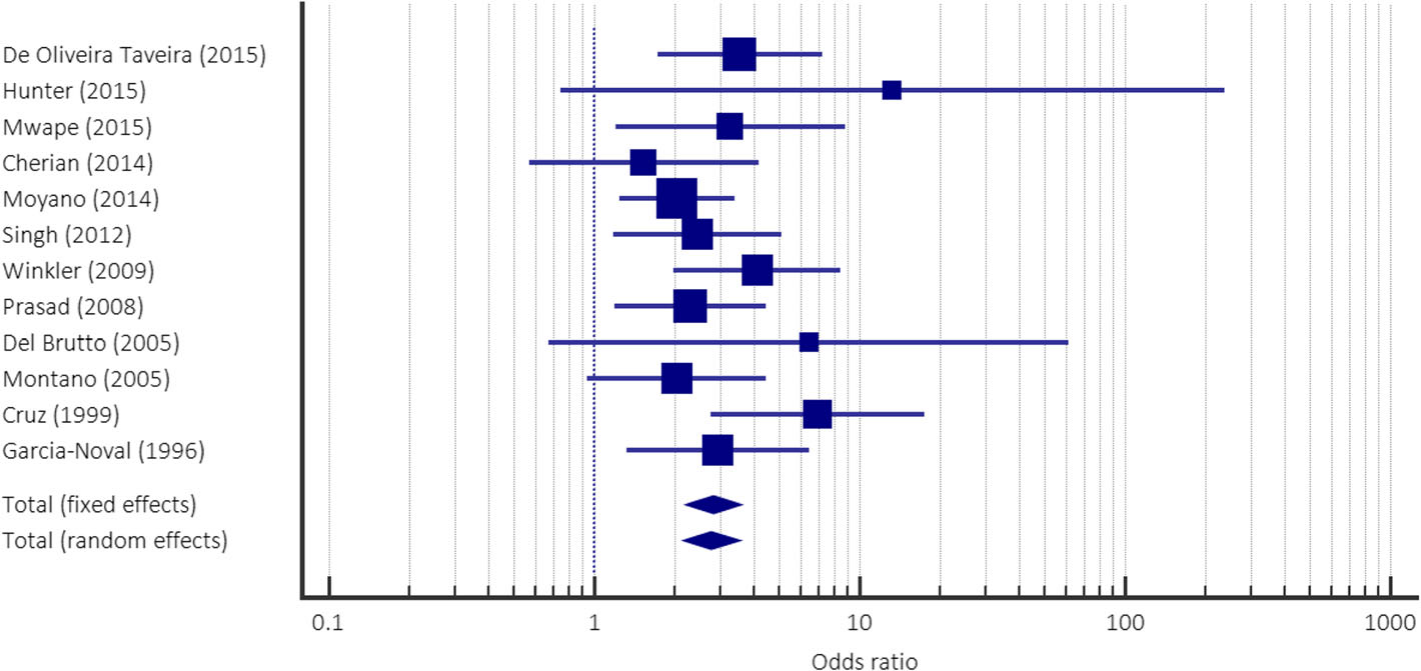
Meta-analysis of case-control studies, showing the individual odds ratios (*OR*s), with associated Cis

Statistical analysis of the remaining 12 case-control studies is shown in Table 1. A statistically significant association (*p* < 0.05) between NCC infection and epilepsy was found in 8 studies, with 2 further studies approaching statistical significance [32, 33]. *OR*s ranged from 2.06 (95% *CI*: 1.25–3.40) to 6.93 (95% *CI*: 2.74–17.51). A statistically significant common *OR*, estimated using a random effects model, of 2.76 (95% *CI*: 2.19–3.48) was found (Fig. 2). This implies that an individual who is exposed to NCC infection has an approximately 2.76 times higher risk of developing epilepsy compared to an individual who is not exposed to NCC infection.

**Table 1:** Results from case-control studies investigating the association between NCC exposure and epilepsy

Study	PWE with NCC (*n*)	PWE without NCC (*n*)	PWOE with NCC (*n*)	PWOE without NCC (*n*)	Odds ratio	95% confidence interval	z-Statistic	*P* value
De Oliveira Taveira et al. (2015) [17]	37	82	12	94	3.54	1.73–7.23	3.46	0.0005
Hunter et al. (2015) [32]	6	170	0	174	13.31	0.74–238.02	1.76	0.0786
Mwape et al. (2015) [40]	20	29	7	33	3.25	1.20–8.79	2.323	0.0202
Cherian et al. (2014) [58]	12	68	7	61	1.54	0.57–4.16	0.848	0.3962
Moyano et al. (2014) [35]	109	173	26	85	2.06	1.25–3.40	2.830	0.0046
Singh et al. (2012) [59]	27	79	13	93	2.45	1.18–5.06	2.412	0.0159
Winkler et al. (2009) [60]	38	174	10	188	4.11	1.99–8.49	3.811	0.0001
Prasad et al. (2008) [41]	29	31	31	76	2.29	1.19–4.42	2.479	0.0132
Del Brutto et al. (2005) [61]	5	14	1	18	6.43	0.67–61.47	1.615	0.1062
Montano et al. (2005) [33]	15	24	26	85	2.04	0.94–4.46	1.794	0.0727
Cruz et al. (1999) [34]	14	12	17	101	6.93	2.74–17.51	4.095	<0.0001
Garcia-Noval et al. (1996) [38]	36	40	12	39	2.93	1.33–6.43	2.669	0.0076

The proportion of PWE with concurrent NCC infection was highly variable, with a range of 36.59% [33] to 100% [32]. It should be noted that, despite NCC being defined as the exposure, and epilepsy as the outcome, both case and control populations were initially defined through the identification of people with or without epilepsy, and NCC diagnosis was determined secondarily. This meant that in the majority of studies, the number of NCC cases, and therefore the size of the exposure groups, were far smaller than the number of epilepsy cases, forming the outcome groups. For example, in Hunter et al., [32] the number of people identified as having NCC was only 6, all of which had epilepsy, giving a proportion of 100% and forming the upper boundary of the proportion range. In contrast, 344 individuals did not have NCC, forming the control group; these two populations are so different in size that comparisons are unlikely to be reliable, which highlights an important limitation evident in multiple studies included for analysis. The proportion of controls with epilepsy was only slightly more consistent, and ranged from 10.62% [34] to 67.05% [35].

The I^2^ test for heterogeneity gave a value of 0.00% (95% *CI*: 0.00–57.84), suggesting a high level of homogeneity between studies, and that differences in study data were not attributable to variations between studies.

### Proportions of NCC in epileptic populations

A total of 26 studies reporting prevalence rates of epilepsy and NCC contained suitable data for the determination of proportions of NCC in epileptic populations (Table 2). Five studies were conducted in Africa, 8 in Asia (all from India), 12 in South America and 1 in Central America. In Mwanjali et al. [36], the only PWE who underwent CT scanning were those who had previously tested positive on Ag-ELISA, to conserve resources for those at higher risk of actually having NCC. However, this meant that out of a total of 123 people identified as having epilepsy, only 28 had a CT scan; this was considered sufficiently low to exclude this study from further statistical analysis. This represents a significant limitation of this review that was encountered quite consistently, where even studies that did have access to neuroimaging tended to rely more strongly on serological data for NCC diagnosis.

**Table 2:** Results from cross-sectional and cohort studies, reporting on prevalence rates of NCC and epilepsy respectively in different populations, with associated proportions

Study	Study country	Number of PWE	Number of people with NCC (Number who had CT or MRI)	Proportion of NCC in PWE	95% confidence intervals
Mwape et al. 2015 [40]	Zambia	56	20 (49)	40.82	27.00–55.79
Sahu et al. 2014 [39]	India	61	23 (61)	37.71	25.61–51.04
Bianchin et al. 2014 [62]	Brazil	191	71 (191)	37.17	30.31–44.45
Moyano et al. 2014 [35]	Peru	301	109 (282)	38.65	32.94–44.61
Millogo et al. 2012 [42]	Burkina Faso	72	20 (68)	29.41	18.98–41.71
Del Brutto and Del Brutto, 2012 [63]	Ecuador	431	120 (431)	27.84	23.66–32.33
Blocher et al. 2011 [64]	Tanzania	212	35 (212)	16.51	11.78–22.21
Goel et al. 2011 [65]	India	141	49 (141)	34.75	26.94–43.22
Raghava et al. 2010 [66]	India	116	39 (101)	38.61	29.09–48.82
Foyaca-Sibat et al. 2009 [23]	South Africa	244	34 (92)	36.96	27.12–47.66
Lescano et al. 2009 [9]	Peru	42	23 (42)	54.76	38.67–70.15
Rajshekhar et al. 2006 [67]	India	194	55 (162)	33.95	26.71–41.80
Velasco et al. 2006 [68]	Brazil	512	139 (512)	27.15	23.34–31.22
Singh et al. 2006 [69]	India	525	169 (525)	32.19	28.21–36.37
Da Gama et al. 2005 [70]	Brazil	89	50 (89)	56.18	45.25–66.68
Nicoletti et al. 2005 [57]	Bolivia	124	34 (105)	32.38	23.57–42.21
Montano et al. 2005 [33]	Peru	39	15 (39)	38.46	23.36–55.38
Del Brutto et al. 2005 [61]	Ecuador	24	5 (19)	26.32	9.15–51.20
Cruz et al. 1999 [34]	Ecuador	31	14 (26)	53.85	33.94–76.48
Palacio et al. 1998 [71]	Colombia	643	76 (546)	13.92	11.13–17.11
Singhi & Singhi, 1997 [54]	India	100	13 (100)	13.00	7.11–21.20
Nair et al. 1997 [72]	India	198	20 (198)	10.10	6.28–15.17
Garcia-Noval et al. 1996 [38]	Guatemala	76	36 (76)	47.37	35.79–59.16
Arruda et al. 1991 [73]	Brazil	210	57 (210)	27.145	21.25–33.69
Gulati et al. 1991 [74]	India	170	27 (170)	15.88	10.74–22.26

**Fig. 3:**
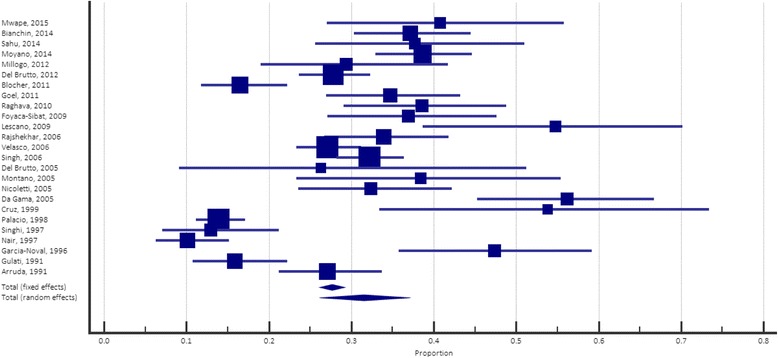
Meta-analysis of cross-sectional and cohort studies, showing the individual proportions of NCC in PWE, and associated 95% *CI*s

Proportions of NCC cases within epileptic populations ranged from 10.10% (95% *CI*: 6.28–15.17) to 56.18% (45.25–66.68). A common proportion of 31.54% (95% *CI*: 26.86–36.41) was estimated using a random effects model; this value suggests that in a population of individuals with epilepsy, approximately one-third of them will have associated NCC infection. However, the I^2^ test for heterogeneity was 91.01% (95% *CI*: 87.98–93.28), suggesting extremely high variability between studies; this makes it relatively unlikely that conclusions drawn from these data will be sufficiently reliable. The forest plot shown in Fig. 3 depicts these results, and also gives a visual representation of the degree of heterogeneity between studies.

There was some variation between proportions when stratified by continent: the mean proportion of NCC-associated epilepsy in African studies was 30.93%, 27.02% in Asian studies, and 36.15% in South American studies. Although none of these results deviate significantly from the common mean, it is worth noting that there is a difference of nearly 10% between Asian and South American studies, suggesting a relatively substantial difference in the strength of association between NCC and epilepsy between these continents. These results therefore suggest a greater proportion of epilepsy cases in South American countries is associated with NCC compared to other endemic areas around the world.

## Discussion

In total, 33 studies met the criteria for inclusion in both statistical and qualitative analyses. A significant association was found between NCC exposure and epilepsy in a meta-analysis of these data, with a common *OR* of 2.76 (95% *CI*: 2.19–3.48) indicating just under a 3 times higher risk of developing epilepsy in individuals infected with NCC compared to those with no NCC infection. This is a slightly lower value compared to a previous systematic review by Quet et al. [37], where a common *OR* of 3.4 (95% *CI*: 2.7–4.3) was reported following meta-analysis of data from studies in Africa only. This study did not specify neuroimaging as an inclusion criterion, instead relying almost exclusively on serological data. Considering Brazil and Ecuador ﻿[38, 39] have the highest reported rates of NCC-epilepsy co-morbidity, it was expected that the inclusion of studies from this continent would have resulted in a higher common *OR* compared to African countries only.

One single case-control study by De Oliveira Taveira et al. [17] investigated the link between NCC and a specific type of epilepsy caused by hippocampal sclerosis, known as mesial temporal lobe epilepsy (MTLE-HS), nested within a study of the association between NCC and epilepsy in general. Results showed a statistically significant association between NCC and MTLE-HS, however when MTLE-HS cases were not included in further analysis, there was no significant association found between NCC and other types of epilepsy.

Another interesting observation made by a number of case-control studies was the high rates of calcified cysticerci in epilepsy, compared to other stages of cyst evolution, such as viable or transitionary phases. Mwape et al. [40] reported that the majority of NCC lesions they identified in PWE were calcified, indicating that seizures are more likely to develop in the later stages of the disease. Furthermore, Prasad et al. [41] noted that asymptomatic NCC cases were more likely to display cysts in the viable stage than in any other stage of NCC development. This was to be expected, as it has been suggested that seizures may be associated with inflammatory responses generated in response to cyst degeneration, whereas viable cysts generally remain undetected due to immune evasion mechanisms [7]. However, despite this general trend, it has been observed that amongst asymptomatic NCC sufferers there exists substantial variability in the different stages of cyst development observed [41], and it is still not certain why some individuals develop symptoms whilst other with similar pathological presentations do not.

A common proportion of NCC cases within epileptic populations of 31.54% (95% *CI*: 26.86–36.41) was estimated using a random effects model; this value indicates that within the populations investigated, approximately one-third of PWE had concurrent NCC infection. This is in accordance with a previous review by Ndimubanzi et al [29], which reported a pooled estimate of 29.0% (95% *CI*: 22.9–35.5%). Results for I^2^ tests of heterogeneity, both in this review and Ndimubanzi et al. [29], were high: 91.01% (95% *CI*: 87.98–93.28) and 92.5% (95% *CI*: 88.1–94.6%) respectively. This highlights a significant amount of between-study variation, which makes it difficult to draw accurate conclusions from associated data. This variation can be attributed to a wide variety of causes, such as differences in study design, use of hospital-based versus community-based populations, and of course the substantial differences in community demographics, such as socioeconomic status, sanitation infrastructure and agricultural practices.

As in the case-control studies, multiple cohort and cross-sectional studies, reported a greater proportion of calcified cysticerci than other phases of cyst development in PWE found to have NCC. In accordance with this finding, the correlation between serology and development of epilepsy was further weakened, due to the fact that serological assays such as EITB only have adequate sensitivity for viable cysticerci, and for the most part do not detect calcified cysts [32]. Interestingly, Millogo et al. [42] found that PWE with NCC were significantly older than PWE without NCC, and therefore hypothesised that the increased proportion of calcified cysts identified may be associated with a correlation between age and symptomatic NCC. These findings may indicate that seizures develop during the later stages of NCC development. However, this correlation has not been sufficiently established, and studies such as those conducted by Mwape et al. [40] found no such association between symptomatic NCC and age.

### Could a genetic predisposition exist?

It cannot be disputed that there are noticeably high rates of comorbidity between NCC and epilepsy in certain areas of the world. While strong evidence to suggest NCC as a direct cause of epilepsy may be lacking, there is a link between the two conditions that still remains to be defined. One hypothesis is that NCC and epilepsy are indirectly linked by a third factor, which may be genetic in nature [43]. That the clinical manifestations of NCC infection may be related to specific genotypes has been under consideration since it became apparent that certain individuals of the same age and sex, within similar populations and with similar pathological findings, can present with contrasting clinical profiles [33].

One study found a significant association between symptomatic individuals experiencing seizures and the expression of MMP-9 polymorphisms leading to increased blood-brain-barrier (BBB) permeability, compared to asymptomatic control subjects [44]. MMP-9 is an enzyme that has a central role in the degradation and breakdown of the BBB, and increased expression may lead to an influx of immune cells that mediate the inflammatory responses and have been associated with the degradation of NCC cysticerci, stimulating seizure activity in affected individuals [44]. This suggests that the propensity to develop symptoms associated with NCC may be related to the degree of inflammation generated in response to parenchymal cysticerci, with certain genetic polymorphisms playing a role in associated regulatory pathways. Another study found an association between polymorphisms of the TLR4 gene and susceptibility to NCC [45]. It has been suggested that the Th2 branch of the adaptive immune response is responsible for maintaining an asymptomatic state in NCC patients, and that a shift from this response to the Th1 response is a key component of the transition from asymptomatic infection to the development of symptoms such as seizures [45]; it was considered a distinct possibility that the polymorphisms identified in this study may drive this shift from the Th2 to the Th1 response.

This theory of genetic involvement in NCC symptomatology is supported by studies reporting familial aggregation of disease phenotypes within certain regions [19, 46]. A study in Mexico found that amongst populations of women who were equally exposed to NCC infection, children born to mothers with a multi-cyst infection had a significantly higher reported risk of also developing a multi-cyst infection, compared to children born to mothers with only single cyst infection, or with no NCC infection [47], suggesting a genetic role in susceptibility to infection. NCC pathologies also show a degree of regional specificity, where localization of the infection to the subarachnoid space is common in Latin America, whereas parenchymal infection is far more prevalent in Asia and Africa [19] which may indicate a correlation between human genotypes and clinical manifestations of the disease. However, other studies report no discernible association between genotype and development of seizures [48].

### A potential link with hippocampal sclerosis

NCC may be involved as a component of a complex pathological pathway that eventually leads to the development of epilepsy, without directly causing individual seizure events. For example, it has been observed that in NCC-endemic countries, there are frequently high rates of individuals diagnosed with mesial temporal lobe epilepsy associated with hippocampal sclerosis (MTLE-HS) who are subsequently found to have a concurrent NCC infection [49]; this could represent a pathway precipitated by NCC infection, in which hippocampal sclerosis functions as an intermediate step in the indirect development of epilepsy. Initial precipitating injuries (IPIs) such as cranial trauma, neonatal hypoxia, and certain infections have already been well documented as indirectly causing temporal lobe epilepsy through hippocampal insult and subsequent sclerosis [50]; it is not inconceivable that a neuroparasitic disease such as NCC could function in a similar way.

The mechanisms behind this hypothetical relationship may either be directly caused by the physical presence of cysticerci within the hippocampal region, leading to inflammation-mediated pathology, or indirectly by the damage and consequent synaptic reorganization caused by repetitive provoked seizures [51]. NCC would function as an IPI in this situation by initially causing provoked symptomatic seizures, but initiating longer-term epileptogenic pathology whereby unprovoked epileptic activity would persist even following the removal of NCC as a stimulus.

Although issues similar to those encountered when assessing the causal nature of the NCC-epilepsy relationship are also present when considering a link with hippocampal sclerosis, a number of studies have reported results that support this theory. Bianchin et al. [52] found that 42.85% of individuals presented with a single parenchymal NCC lesion that was directly associated with the physiological location of MTLE-HS, suggesting an anatomical relationship between the two conditions; this indicates reasonable evidence to refute the possibility of coincidental comorbidity. De Oliveira Taveira et al. [17], found a statistically significant association between NCC and epilepsy only when cases of MTLE-HS were included in the meta-analysis, however when these cases were excluded, there was no association found between NCC and other types of epilepsy. Data from studies investigating the link between NCC and epilepsy could appear to clearly show a significant association between the two, but with a different underlying link existing, that connects them, indirectly. Other studies indicate a coincidental relationship only between NCC and MTLE-HS [53], probably due to high prevalence rates of both conditions in certain endemic countries.

Despite trying to avoid the major limitations encountered by previous studies and reviews, there were some issues that occurred consistently throughout the study selection and analysis processes. Population sizes were often initially very large, where substantial proportions of certain communities were screened for the presence of either NCC or epilepsy, but the study would identify only very small numbers of individuals who were afflicted with either condition, therefore being relevant to the investigation. Data sets for a number of studies were much smaller than anticipated, which led to greater heterogeneity between papers. It was also very difficult to avoid study selection bias in terms of the actual populations that were sampled, since in order to identify a great enough number of individuals with NCC to make the study worthwhile, endemic populations, often of a very particular type, were specifically chosen. For example, a large number of studies sampled pig farming communities only, however by doing so, significant selection bias is introduced, and the studies cannot be extrapolated to the general population. The use of hospital-based studies, as opposed to community-based studies may also have introduced selection bias.

Despite applying strict exclusion criteria regarding the use of neuroimaging, the majority of included studies, all with access to CT or MRI, tended to rely far more heavily on serological data for the diagnosis of NCC (see references in Tables 1 and 2). While this is more practical in rural communities in endemic countries, this can lead to significant overestimations of the number of individuals with active NCC infection. Several studies had a limited number of CT scans available to them due to financial restrictions, so these would only be carried out as confirmatory tests on random samples of serologically positive individuals, or would only be used on those deemed at highest risk of having the infection. Neuroimaging is generally considered a more reliable diagnostic technique than serological assays, as NCC infection can be directly visualised within the brain, and cysts can be accurately localised to specific areas. Ideally, all study subjects would receive some form of neuroimaging, results from which would form the most substantial evidence for the diagnosis of NCC, with serology serving only as further confirmation.

However, despite the emphasis placed on the benefit of using neuroimaging it must be noted that they also have flaws that may have led to the misclassification of NCC cases in some studies. Although certain aspects of a lesion identified through neuroimaging may display features that strongly indicate an NCC infection, such as an identifiable scolex [30], lesions that are not so clearly defined could be attributed to other infectious causes, such as tuberculosis (TB), or, rarely, tumours or microabscesses [54]. TB is difficult to distinguish from NCC, especially in countries where there are very high prevalence rates of both diseases [55]. Although guidelines are available to help differentiate TB and NCC when assessing results from CT or MRI, they are not infallible [55]. Administration of the Mantoux test and chest X-rays may help to exclude TB as a differential diagnosis in study participants [56], but this approach was not adopted by the majority of studies and it cannot be certain that all lesions identified as NCC were not of a different pathological origin.

Finally, there was a dearth of studies reporting incidence, as opposed to prevalence data. A major issue with using epilepsy prevalence data is that there is no way of determining whether NCC (as exposure), occurred before or after the development of epilepsy, the proposed outcome [57]. This makes it very difficult to assess whether or not the epilepsy was caused by the NCC, or whether it was a pre-existing condition. The ideal design for a study assessing causality would be a prospective study in which a cohort of newly-diagnosed NCC sufferers are followed up over a number of years to see how many subsequently develop epilepsy.

## Conclusion

While our results from this review concur with previous investigations in concluding that NCC does increase the risk of developing epilepsy, and has a reasonably high prevalence in populations of PWE in certain endemic countries, and it cannot be disputed that there are high rates of comorbidity in certain populations, existing available data are not sufficient to determine the existence of a causal association between NCC and epilepsy. To understand the complex interplay between these two conditions, a number of changes to the way in which research in this area is carried out need to be implemented. Study designs should be prospective in nature to accurately determine epilepsy incidence in cohorts of NCC sufferers, so that it can be clearly stated that epilepsy is an outcome of NCC, and does not represent a pre-existing condition due to high prevalence rates of both diseases in endemic countries. In addition to this, a universal set of diagnostic criteria, such as those proposed by Del Brutto et al. [30], should be consistently implemented across all studies, so that accurate comparisons can be made between data collected in the majority of studies worldwide. Improvements to future studies will also depend directly on the increased availability of resources such as CT and MRI in developing endemic countries. In the absence of reliable estimates of its global burden, NCC will remain of low priority in the eyes of funding agencies - a truly neglected disease.

## Additional files

Additional file 1: Multilingual abstract in the six official working languages of the United Nations. (PDF 712 kb)

Additional file 2: Characteristics of case-control studies. (DOCX 115 kb)

Additional file 3: Characteristics of cross-sectional and cohort studies. (DOCX 108 kb)

## Abbreviations

Ag-ELISA: Antigen enzyme-linked immunosorbent assay
BBB: Blood-brain-barrier
CNS: Central nervous system
CT: Computed tomography
ICP: Intra-cranial pressure
IPIs: Initial precipitating injuries
MRI: Magnetic resonance imaging
MTLE-HS: Mesial temporal lobe epilepsy with hippocampal sclerosis
NCC: Neurocysticercosis
*OR*s: Odds ratios
PWE: People with epilepsy
TB: Tuberculosis
