# Supplementary material for: The causal relationship between neurocysticercosis infection and the development of epilepsy - a systematic review
Source: Infect Dis Poverty. 2017 Apr 5;6:31. doi: 10.1186/s40249-017-0245-y (PMC5381143; doi:10.1186/s40249-017-0245-y)
Supplement: Supplementary file 1 — Multilingual abstract in the six official working languages of the United Nations. (PDF 712 kb) [file 40249_2017_245_MOESM1_ESM.pdf]

Translation of the abstract into the six official working languages of the United Nations

العلاقة السببية بين الإصابة بداء الكيسات المذنبة والإصابة بمرض الصرع – مراجعة منهجية

لوسي جريير وسوزان سي ويلبورن

ملخص
خلفية: داء الكيسات المذنبة هو مرض طفيلي يصيب الجهاز العصبي المركزي للإنسان، الشكل الأكثر شيوعاً منه هو ذلك الذي يصيب نسيج الدماغ بالشكل اليرقي للدودة الشريطية الوحيدة. العلاقة السببية بين الإصابة بداء الكيسات المذنبة وتفاقم مرض الصرع لدى الأفراد المصابين به هو أمر مسلم به، مدعوم في جزء منه بمستويات عالية من التواكب المرضيّ في البلدان الموبوءة في العالم.
طرائق: قامت هذه الدراسة باعتماد مراجعة منهجية وتحليل انتقادي حول العلاقة بين داء الكيسات المذنبة ومرض الصرع مع هدف أولي يتمثل في تحديد كمية الخطر الناجم عن الإصابة بالصرع المتبوع بالإصابة بداء الكيسات المذنبة. وهدف ثانوي يتمثل في تحليل نسب الإصابة بداء الكيسات المذنبة المرافق لمرض الصرع لدى شعوب مختلفة. تم التشديد كثيراً على أهمية توافر التصوير العصبي (التصوير المقطعي والتصوير بالرنين المغناطيسي) واستخدام أدلة توجيهية واضحة لتشخيص مرض الصرع، في سبيل تفادي التقديرات المبالغ بها للنسب الشائعة حول كل حالة، تم تحديد قيد في عدة دراسات سابقة.
نتائج: المعدلات الشائعة 2.76 التي تم تحديدها من التحليل التلوي لدراسة عدة حالات، أشارت إلى أن الفرد المصاب بداء الكيسات المذنبة معرض بأكثر من ثلاث مرات لخطر الإصابة بمرض الصرع من الفرد غير المصاب. علاوة على ذلك، فإن دراسات التحليل التلوي حددت نسبة شائعة تبلغ 31.54% من حالات الإصابة بالصرع المترافقة مع داء الكيسات المذنبة وهو ما يشير إلى أن ثلث المصابين بالصرع في البلدان المعرضة للمخاطر، مصابون بداء الكيسات المذنبة كذلك.
خلاصة: من النتائج المهمة هو نقص البيانات السريرية الجيدة للوصول إلى تحديد دقيق للعلاقة السببية. حتى الدراسات التي تم تضمينها تحتوي على قيود ملحوظة، بما فيها النقص العام في ثبات التشخيص ونقص البيانات الدقيقة المتعلقة بالأوبئة. هذه المراجعة تضيء على الحاجة للثبات في البحث في هذا المضمار. في ظل غياب التقديرات التي يمكن الاعتماد عليها عن عبئه العالمي، فإن داء الكيسات المذنبة يحظى بأولوية منخفضة في نظر وكالات التمويل حيث يعتبر مرضاً مهملاً بحق.

Translated from English version into Arabic by sjaatoul, through

TRANSLATORS
WITHOUT BORDERS

神经囊尾蚴病与癫痫的因果关系：系统评价

Lucy Gripper and Susan C Welburn

摘要
引言：神经囊尾蚴病（NCC）是由于猪带绦虫幼虫侵入人体中枢神经系统，最常见的是累及脑实质。人们已经认识到 NCC 感染与受感染者的癫痫发病之间的因果关系，世界上流行国家二者的高度共发病在一定程度上支持这一病因关系。
方法：本研究对 NCC 和癫痫关系进行系统综述和关键性分析。主要目的是定量评价罹患 NCC 后发生癫痫的风险，次要目的是分析不同人群中 NCC 相关癫痫所占的比例。本研究强调使用神经影像学（CT 或 MRI）和使用明确的癫痫诊断指南的重要性，以避免高估任一疾病的患病率；我们发现这一点是以前几项研究的一大缺陷。
结果：对病例对照研究的 meta 分析得出了合并的比值比为 2.76，表明 NCC 患者罹患癫痫的风险比未感染者高出近三倍。此外，meta 分析显示 31.54%癫痫病例与 NCC 有关，这表明在流行国家的癫痫患者中，近三分之一可能与 NCC 有关。
结论：一个重要发现是缺乏好的临床数据来准确确定二者的因果关系。纳入的研究存在明显的局限性，如总体上缺乏诊断方面的一致性和缺乏准确的流行病学数据。本综述强调了这一领域研究对一致性的需求。在缺乏对其全球负担可靠估计的情况下，NCC 在资助机构那里仍将是低优先级的，而成为真正被忽视的疾病。

Translated from English version into Chinese by Men-Bao Qian, edited by Pin Yang

CDC CHINESE CENTER FOR DISEASE CONTROL AND PREVENTION
NATIONAL INSTITUTE OF PARASITIC DISEASES

Lien de cause à effet entre neurocysticercose et apparition de l’épilepsie: une revue systématique

Lucy Gripper et Susan C Welburn

Résumé
Contexte: La neurocysticercose est une parasitose du système nerveux central humain qui résulte, sous sa forme la plus fréquente, de l'infestation du parenchyme cérébral par la forme larvaire de *Taenia solium.* La relation de cause à effet entre neurocysticercose et apparition d'une épilepsie chez les sujets infectés est admise, en partie en raison de la fréquence de cette association dans les pays d'endémie du monde entier.
Méthodes: La présente étude est une revue systématique et une analyse critique du lien entre neurocysticercose et épilepsie, dont le but premier est de quantifier le risque d'apparition d'une épilepsie à la suite d'une infestation du système nerveux par les cysticerques. Un but secondaire de l'étude était d'analyser les proportions de cas d'épilepsie associés à la neurocysticercose dans différentes populations. Il ressort de cette revue que la disponibilité d'équipements de neuro-imagerie (TDM ou IRM) et l'application de lignes directrices claires pour le diagnostic de l'épilepsie constituent deux conditions importantes pour éviter la surestimation de la prévalence de l'une ou l'autre des deux pathologies, qui constitue une faiblesse de plusieurs études antérieures.
Résultats: La méta-analyse d'études cas-témoin a donné un rapport de cotes commun de 2,76, qui indique qu'un sujet atteint de neurocysticercose a presque trois fois plus de risques de développer une épilepsie qu'un sujet non infesté. En outre, la méta-analyse des études a mis en évidence une association de 31,54 % des cas d'épilepsie à une neurocysticercose, ce qui suggère qu'un tiers environ de la population épileptique des pays à risques peut être infectée par le parasite.
Conclusion: Une observation importante concerne le manque de bonnes données cliniques susceptibles de permettre une détermination précise d'un lien de cause à effet. Les études que nous avons incluses ne sont pas non plus exemptes de limitations, notamment parce que l'approche diagnostique n'est pas homogène et que l'on manque de données épidémiologiques exactes. Notre revue souligne la nécessité d'une harmonisation des recherches dans ce domaine. En l'absence d'estimations sûres de son fardeau mondial, la neurocysticercose restera une maladie véritablement négligée, loin des priorités des bailleurs de fonds.

Translated from English version into French by Suzanne Assenat, through

TRANSLATORS
WITHOUT BORDERS

Причинно-следственная связь между нейроцистицеркозом и развитием эпилепсии: систематический обзор

Люси Гриппер (Lucy Gripper) и Сьюзан Велбёрн (Susan C Welburn)

Аннотация
Краткое описание. Нейроцистицеркоз (НЦЦ) – это паразитарная инфекция центральной нервной системы человека, наиболее распространённая форма которой приводит к заражению паренхимы головного мозга личинками ленточного червя Taenia solium. Установлена причинно-следственная связь между этим видом НЦЦ-инфекции и развитием эпилепсии у инфицированных лиц, и это частично подтверждается высокими уровнями коморбидности в эндемичных странах во всем мире.
Методы. В настоящем исследовании были проведены систематический обзор и критический анализ взаимосвязи между НЦЦ и эпилепсией, при этом главной задачей являлось количественная оценка риска развития эпилепсии после НЦЦ-инфекции. Вторичная цель исследования заключалась в определении процентных долей заболеваемости эпилепсией вследствие заражения НЦЦ-инфекцией среди различных популяций. Большое внимание уделялось доступности к нейровизуализационному обследованию (КТ или МРТ) и применению чётких инструкций для диагностирования эпилепсии, чтобы избежать завышенных оценок показателей распространённости обоих заболеваний. Подобное наблюдалось в нескольких предыдущих исследованиях.
Результаты. На основании мета-анализа исследований типа «случай-контроль» было установлено, что общее отношение шансов равно 2,76. Это означает, что у больного, зараженного НЦЦ, риск развития эпилепсии почти в три раза выше, чем у незараженного человека. Помимо этого, мета-анализ исследований выявил 31,54% заболеваний эпилепсией, связанных с НЦЦ-инфекцией. Это позволяет предположить, что среди эпилептических популяций в странах с высокой степенью риска примерно одна треть заболеваний может быть связана с НЦЦ-инфекцией.
Заключение. Важным результатом явилось обнаружение факта отсутствия хороших клинических данных, позволяющих точно определить причинно-следственную связь. Даже проанализированные исследования имели

заметные ограничения, включая недостаток единого подхода при диагностировании в целом и нехватку достоверных эпидемиологических данных. Данный обзор подчёркивает необходимость согласованных исследований в этой области. В отсутствии надёжных оценок глобальных последствий НЦЦ будет продолжать оставаться низкоприоритетным для финансирующих организаций и поистине обделённым вниманием заболеванием.

Translated from English version into Russian by Natalia Potashnik, through

TRANSLATORS
WITHOUT BORDERS

Efectos adversos relacionados con la terapia antiretroviral: Puede el África sub Sahariana hacer frente a la nueva política de "prueba y tratamiento" de la Organización Mundial de la Salud?

Jobert Richie N. Nansseu, Jean Joel R. Bigna

Resumen
Antecedentes: Recientes estudios han demostrado que el inicio temprano de una terapia antiretroviral (TARV) tiene como resultado una significativa reducción en la transmisión del VIH. Este es el motivo principal detrás de la política de "prueba y tratamiento de la "Organización Mundial de la Salud (OMS). La implementación de esta política creará a una mayor incidencia de los efectos adversos relacionados con TARV, - en África Sub Sahariana (SSA). Está ya lista la región para hacer frente a tal desafío?
Cuerpo Principal: La introducción y el amplio uso de la TARV ha cambiado de manera drástica la historia natural del VIH/SIDA, pero la exposición a la TARV lleva a serios efectos adversos relacionados con la medicación que se explican principalmente por la toxicidad mitocondrial y la situación empeorará en el futuro próximo. De hecho, la TARV está asociada con un mayor riesgo de desarrollar enfermedades cardiovasculares, lipodistrofia, pre diabetes y diabetes manifiesta, resistencia a la insulina e hiperlactatemia/ acidosis láctica. La prevalencia de estos trastornos ya es alta en SSA, y la situación empeorará debido a la implementación de las nuevas recomendaciones de la OMS. La mayoría de los países del SSA se caracterizan por extrema pobreza, sistemas de salud muy débiles, servicios de salud de baja calidad e inadecuados, inaccesibilidad a las instalaciones de salud existentes, falta de personal de salud calificado, falta de equipos adecuados, medicinas inaccesibles e inasequibles y carga pesada en un contexto de la doble carga de la enfermedad. Adicionalmente, en la SSA hay escases de datos sobre la incidencia y factores predictivos de los afectos adversos que se relacionan con la TARV, para anticipar estrategias que deben implementarse para prevenir que se presenten estas condiciones o estimar adecuadamente la futura carga y preparar un plan de respuesta que sea adecuado. Esto se requiere si queremos anticipar y prevenir de forma efectiva esta futura carga.
Conclusión: Pese a que SSA sería la primera región que experimente los inmensos beneficios de implementar la política de "prueba y tratamiento" de la OMS, la región no está aún preparada para manejar el peso mayor como consecuencia de las complicaciones metabólicas y tóxicas relacionadas con la TARV. Se deben tomar medidas urgentes para llenar los vacíos si no se quiere que la SSA se vea sobrecargada por las consecuencias de la política de "prueba y tratamiento".

Translated from English version into Spanish by Doloritas, through

TRANSLATORS
WITHOUT BORDERS
