# Supplementary material for: The causal relationship between neurocysticercosis infection and the development of epilepsy - a systematic review
Source: Infect Dis Poverty. 2017 Apr 5;6:31. doi: 10.1186/s40249-017-0245-y (PMC5381143; doi:10.1186/s40249-017-0245-y)
Supplement: Supplementary file 2 — Characteristics of case-control studies. (DOCX 115 kb) [file 40249_2017_245_MOESM2_ESM.docx]

| **General Information** | | | | **Cases (PWE)** | | | | **Controls (PWOE)** | | **Detection of NCC** | |
| --- | --- | --- | --- | --- | --- | --- | --- | --- | --- | --- | --- |
| **Authors** | **Year of Publication** | **Study Design** | **Study Country** | **Source** | **Age** | **Classification of Epilepsy** | **Methods of Diagnosis** | **Source** | **Matching Criteria** | **Methods of Diagnosis** | **Criteria** |
| De Oliveira Taveira *et al.*  [17] | 2015 | Case-control | Brazil | University of Campinas Hospital. | >15 years | ILAE, 2010.  http://www.ilae.org | Epilepsy diagnosis previously established. Medical records reviewed. | University of Campinas Hospital | Similar socioeconomic background. | High resolution MRI and CT scan. | Del Brutto’s diagnostic criteria (2001).  [30] |
| Hunter *et al.*  [32] | 2015 | Case-control | Tanzania | General population. Screening questionnaire used to identify. | Unspecified | Unspecified. | Examination of potential cases, and confirmation of diagnosis by the research doctor. | Randomly selected from census database. | Age, sex and area of residency. | CT scan and western blot analysis for  *T. solium* antibodies. | Del Brutto’s diagnostic criteria (2001).  [30] |
| Mwape *et al.*  [38] | 2015 | Cross-sectional, case-control | Zambia | General population. Screening questionnaire used to identify. | Unspecified | Winkler *et al.* (2007)  [27] | Medical history and a detailed neurological examination. | Consecutive CT scans from the hospital database with clear pathology, negative for NCC. | None. | CT scan, Ag-ELISA and EITB. Also, stool sample analysis with Ag-ELISA. | Del Brutto’s diagnostic criteria (2001).  [30] |
| Cherian *et al*  [56] | 2014 | Case-control | India | Sree Chitra Tirunal Institute for Medical Sciences and Technology. | 18-60 years | Fisher *et al.* (2005)  [28] | Clinical examination, and CT, MRI and/or EEG data reviewed from patient medical records. | Families of epileptic patients. | Sex, age and family. | EITB. Also, CT or MRI was available for 69 out of 80 patients. | Unspecified. |
| Moyano e*t al.*  [35] | 2014 | Cross-sectional, case-control | Peru | General population. Screening questionnaire used to identify. | >2 years | ILAE  http://www.ilae.org | Examination of potential cases, and confirmation of diagnosis by a neurologist. | Archived EITB and CT scan data from two previous population-based studies that estimated NCC prevalence in the general population. | None. | EITB and CT scan. | Unspecified. |
| Singh e*t al.*  [57] | 2012 | Case-control | India | General population. Screening questionnaire used to identify. | Unspecified | ILAE, 1993  http://www.ilae.org | Neurologic assessment by an epileptologist. Sleep and awake EEG examinations and specialised epilepsy-protocol MRI. | Randomly selected from the general population. | Sex and age. | MRI and EITB. | Del Brutto’s diagnostic criteria (2001).  [30] |
| Secka e*t al.*  [31] | 2010 | Case-control | Gambia | Random selection from patients at the Royal Victoria Teaching Hospital or other outpatient clinics and health centres. | Unspecified | ILAE, 1989  http://www.ilae.org | Assessment by the authors using ILAE guidelines. | Randomly selected from the general population. | Sex, age and area of residency. | Ag-ELISA, EITB and CT scan. | Unspecified. |
| Winkler e*t al*  [58] | 2009 | Case-control | Tanzania | The Haydom Lutheran Epilepsy Clinic. | >10 years | Winkler e*t al.* (2007)  [27] | Examination of potential cases, and confirmation of diagnosis by a neurologist. | Patients undergoing CT scans for reasons other than seizures between January 2005 and July 2006. | None. | CT scan, and serum and CSF western blot analysis of anticysticercal antibodies. | Del Brutto’s diagnostic criteria (2001).  [30] |
| Prasad *et al.*  [39] | 2008 | Case-control | India | General population. Screening questionnaire used to identify. | Unspecified | ILAE, 1993  http://www.ilae.org | Clinical evaluation and classification by ILAE guidelines. | Asymptomatic family members of epilepsy cases. | Family. | MRI and EITB. | Del Brutto’s diagnostic criteria (2001).  [30] |
| Del Brutto e*t al.*  [59] | 2005 | Cross-sectional, case-control | Ecuador | General population. Screening questionnaire used to identify. | >15 years | ILAE, 1989  http://www.ilae.org | Examination by two neurologists, and EEG. | Randomly selected from the general population. | Age and sex. | CT scan and EITB. | Del Brutto’s diagnostic criteria (2001).  [30] |
| Montano *et al.*  [33] | 2005 | Cross-sectional, case-control | Peru | General population. Screening questionnaire used to identify. | Unspecified | ILAE, 1989  http://www.ilae.org | Interview and examination by a neurologist. Confirmation of diagnosis obtained from a different physician. | Randomly selected from the general population. | None. | CT scan and EITB. | Unspecified. |
| Cruz e*t al.*  [34] | 1999 | Cross-sectional, case-control | Ecuador | General population. Screening questionnaire used to identify. | Unspecified | ILAE, 1993  http://www.ilae.org | Examination by two neurologists. An awake routine EEG was also performed. | Randomly selected from the general population. | None. | CT scan and EITB. | Unspecified. |
| Garcia-Noval e*t al.*  [60] | 1996 | Cross-sectional, case-control | Guatemala | General population. Screening questionnaire used to identify. | Unspecified | ILAE, 1993  http://www.ilae.org | Examination by a neurologist, and EEG where necessary. | Randomly selected from the general population | None. | CT scan and EITB. | Unspecified. |
