# Supplementary material for: The causal relationship between neurocysticercosis infection and the development of epilepsy - a systematic review
Source: Infect Dis Poverty. 2017 Apr 5;6:31. doi: 10.1186/s40249-017-0245-y (PMC5381143; doi:10.1186/s40249-017-0245-y)
Supplement: Supplementary file 3 — Characteristics of cross-sectional and cohort studies. (DOCX 108 kb) [file 40249_2017_245_MOESM3_ESM.docx]

| **General Information** | | **Study Characteristics** | | | | | **Epilepsy Diagnosis** | | **NCC Diagnosis** | |
| --- | --- | --- | --- | --- | --- | --- | --- | --- | --- | --- |
| **Authors** | **Year of Publication** | **Study Design** | **Study Country** | **Source Population** | **Study Population** | **Age Range** | **Classification of Epilepsy** | **Methods of Diagnosis** | **Methods of Diagnosis** | **Criteria** |
| Mwape *et al*  [38] | 2015 | Cross-sectional, case-control | Zambia | All inhabitants within a 7km radius from the Mtandaza Rural Health Centre. 4443 individuals screened for the presence of epilepsy using a questionnaire. | 51 individuals diagnosed with active epilepsy. | Unspecified | Winkler *et al*. (2007)  [27] | Medical history and a detailed neurological examination. | CT scan, Ag-ELISA and EITB. Also, stool sample analysis with Ag-ELISA. | Del Brutto’s diagnostic criteria (2001) [30] |
| Sahu *et al*  [61] | 2014 | Cohort | India | Paediatric patients of a teaching hospital in Andhra Pradesh Province. | 61 paediatric subjects presenting with afebrile seizure. | 1-15 years | Unspecified | Clinical history taken from each subject or a parent/guardian. Subjects also underwent clinical examination by a neurologist. | Ab-ELISA and CT scan. | Del Brutto’s diagnostic criteria (2001) [30] |
| Bianchin e*t al*  [62] | 2014 | Cohort | Brazil | Patients who were surgically treated for MTLE-HS at the Centre for Epilepsy Surgery at Ribeirao Preto from 1995 to 2000. | 91 consecutive patients diagnosed with MTLE-HS. | Unspecified | Unspecified | Clinical history, neurological examination, and video-EEG analysis. | CT scan and MRI. | Del Brutto’s diagnostic criteria (2001)  [30] |
| Moyano *et al*  [35] | 2014 | Cross-sectional, case-control | Peru | 17,452 individuals inhabiting various communities surrounding the Tumbes River were screened for the presence of epilepsy using a questionnaire. | 301 individuals diagnosed with epilepsy. | >2 years | ILAE  http://www.ilae.org | Examination of potential cases, and confirmation of diagnosis by a neurologist. | EITB and CT scan. | Unspecified |
| Mwanjali *et al*  [36] | 2013 | Cross-sectional | Tanzania | 830 individuals inhabiting 13 randomly selected villages in Mbozi district completed a questionnaire allowing for collection of demographic data and information on clinical signs. | 123 individuals having a history of epileptic seizures. | 15-60 years | ILAE  http://www.ilae.org | Clinical history. | Ag-ELISA, Ab-ELISA and CT scan. Also stool sample analysis with Ag-ELISA. | Unspecified |
| Millogo *et al*  [40] | 2012 | Cross-sectional | Burkina Faso | 888 randomly selected individuals from 3 villages with different pig-farming practices were screened for the presence of epilepsy using a questionnaire. | 39 randomly selected individuals and 33 self-reported cases were confirmed as having epilepsy  (total = 72). | >7 years | ILAE  http://www.ilae.org | Clinical history and full neurological examination. | Ag-ELISA and CT scan. | Modified Del Brutto criteria (2001) [30] where positive Ag-ELISA replaces EITB as a major criterion. |
| Del Brutto & Del Brutto  [63] | 2012 | Cohort | Ecuador | Patients with late-onset epilepsy, attending the Hospital-Clinica Kennedy in Guayaquil. | 431 consecutive patients evaluated between January 1990 and December 2009. | 20-94 years | Unspecified | Clinical history and neurological evaluation. | CT and MRI. | Del Brutto’s diagnostic criteria (2001). [30] |
| Blocher *et al*  [64] | 2011 | Cohort | Tanzania | 346 patients of the Haydom Lutheran Hospital, with a diagnosis of epilepsy, identified in a former project between 2002 and 2004. | 212 epileptic patients who consented to undergo CT scanning. | >10 years | ILAE, 1981  http://www.ilae.org | Clinical history and physical examination. | CT scan and EITB. | Del Brutto’s diagnostic criteria (2001). [30] |
| Goel *et al*  [65] | 2011 | Cross-sectional | India | 14,086 residents of the Chakrata block of Dehradun district were screened for a history of afebrile seizure, using a questionnaire. | 141 patients were confirmed as having two or more unprovoked seizures in the previous 5 years. | Unspecified | ILAE, 1993  http://www.ilae.org | Clinical history, neurological examination and EEG. | CT scan. | Del Brutto’s diagnostic criteria (2001). [30] |
| Raghava *et al*  [66] | 2010 | Cross-sectional | India | 38,105 individuals from 16 randomly selected rural clusters of the Kaniyambadi block of Vellore district were screened for the presence of epilepsy, using a questionnaire. | 166 individuals were diagnosed with active epilepsy. | 2-60 years. | Unspecified | Clinical history and examination by a clinician. | CT scan, EITB and Ag-ELISA. | Del Brutto’s diagnostic criteria (2001). [30] |
| Foyaca-Sibat *et al*  [23] | 2009 | Cohort | South Africa | 296 individuals with suspected epilepsy from the outpatient clinic at St Elizabeth’s hospital in Lusikisiki. | 244 individuals were confirmed as having epilepsy. | 5-76 years | ILAE  http://www.ilae.org | Clinical history. | Ag-ELISA, Ab-ELISA and CT scan. | CT scan positive for NCC-compatible lesions. |
| Lescano e*t al*  [9] | 2009 | Cross-sectional | Peru | 858 residents of 7 rural villages in the district of Matapalo were screened for the presence of epilepsy, using a questionnaire. | 42 individuals were confirmed as having a lifetime history of seizures. | Unspecified | ILAE, 1989  http://www.ilae.org | Clinical history, and evaluation by two different neurologists. | EITB and CT scan. | CT scan positive for NCC-compatible lesions. |
| Rajshekhar *et al*  [67] | 2006 | Cross-sectional | India | 50,617 residents of Vellore district were screened for the presence of epilepsy using a questionnaire. | 194 individuals were diagnosed with active epilepsy. | 2-60 years. | Unspecified | Clinical history and two evaluations by a clinician and a neurologist. | EITB and CT scan. | Del Brutto’s diagnostic criteria 2001. [30] |
| Velasco *et al*  [68] | 2006 | Cohort | Brazil | 512 patients evaluated at the outpatient clinic for intractable epilepsy at the Ribeirao Preto School of Medicine Hospital. | 512 patients with intractable epilepsy. | >18 years | Unspecified | Clinical history, neurological examination and EEG. | CT and MRI. | Del Brutto’s diagnostic criteria 2001. [30] |
| Singh *et al*  [69] | 2006 | Cohort | India | 1026 consecutive patients with history of definite seizures or epilepsy presenting to the adult neurology unit of a teaching hospital in North India between 1995 and 2001. | 398 patients diagnosed with prevalent epilepsy, and 127 with incident epilepsy (total = 525). | >12 years | ILAE, 1993  http://www.ilae.org | Clinical history and EEG. | CT, MRI, EITB and ELISA. | CT scan or MRI positive for NCC-compatible lesions. |
| Da Gama *et al*  [70] | 2005 | Cohort | Brazil | Patients attending the Campinas State University Epilepsy Clinic. | 89 patients were confirmed as having epilepsy, and were divided into those with mesial temporal lobe epilepsy and extra-temporal epilepsy. | 7-62 years | ILAE, 1989  http://www.ilae.org | Clinical history and EEG. | CT and MRI. | CT scan or MRI positive for NCC-compatible lesions. |
| Nicoletti e*t al*  [55] | 2005 | Cross-sectional | Bolivia | 9955 inhabitants of 55 communities in the Cordillera province were screened for the presence of epilepsy using a questionnaire. | 124 individuals diagnosed with prevalent epilepsy. | Unspecified | ILAE, 1981  http://www.ilae.org | Clinical history, neurological examination and EEG. | EITB and CT scan. | Del Brutto’s diagnostic criteria (2001) [30] |
| Montano *et al*  [33] | 2005 | Cross-sectional, case-control | Peru | 903 residents of the district of Matapalo were screened for the presence of epilepsy using a questionnaire. | 39 individuals with a confirmed history of seizures. | Unspecified | ILAE, 1989  http://www.ilae.org | Interview and examination by a neurologist. Confirmation of diagnosis obtained from a different physician. | CT scan and EITB. | Unspecified. |
| Del Brutto *et al*  [59] | 2005 | Cross-sectional, case-control | Ecuador | 2415 residents of the village of Atahualpa were screened for the presence of epilepsy using a questionnaire. | 24 individuals diagnosed with epilepsy. | >15 years. | ILAE, 1989  http://www.ilae.org | Examination by two neurologists, and an EEG. | CT scan and EITB. | Del Brutto’s diagnostic criteria (2001) [30] |
| Cruz *et al*  [34] | 1999 | Cross-sectional, case-control | Ecuador | 2723 inhabitants of the village of San Pablo del Lago were screened for the presence of epilepsy using a questionnaire. | 31 individuals diagnosed with active epilepsy. | Unspecified | ILAE, 1993  http://www.ilae.org | Examination by two individual neurologists. An awake routine EEG was also performed. | CT scan and EITB. | Unspecified. |
| Palacio *et al*  [71] | 1998 | Cohort | Colombia | Individuals with epilepsy who attended any of the 9 outpatient clinics of the Instituto Neurologico de Antioquia between March and October 1995. | 643 individuals diagnosed with epilepsy. | Unspecified | WHO, 1993 | Clinical history and evaluation by a neurologist. | CT scan and EITB. | CT scan positive for NCC-compatible lesions. |
| Singhi & Singhi  [52] | 1997 | Cohort | India | 124 consecutive children presenting with partial seizures to the paediatric emergency service of Nehru Hospital in Chandigarh over a 2-year period. | 100 children with a confirmed history of partial seizures. | 3 months-12 years | ILAE, 1981  http://www.ilae.org | Clinical history, complete physical and neurological examination, and EEG. | CT scan. | CT scan positive for NCC-compatible lesions. |
| Nair *et al*  [72] | 1997 | Cohort | India | Children registered in the neurology clinic and who underwent CT scans for evaluation of simple partial seizures between 1992 and 1993. | 198 children with a confirmed history of simple partial seizures, who underwent CT scans. | Neonatal-15 years | ILAE, 1981  http://www.ilae.org | Clinical records, containing history, and details of neurological and physical examinations were reviewed. | CT scan, and Ab-ELISA on CSF samples. | CT scan positive for NCC-compatible lesions. |
| Garcia-Noval *et al*  [60] | 1996 | Cross-sectional, case-control | Guatemala | 1161 inhabitants of the El Jocote community, and 1131 inhabitants of the Quesada community (total = 2292) were screened for the presence of epilepsy using a questionnaire. | 65 individuals diagnosed with active epilepsy. | Unspecified | ILAE, 1993  http://www.ilae.org | Examination by a neurologist, and EEG where necessary. | CT scan and EITB. | Unspecified. |
| Arruda  [73] | 1991 | Cohort | Brazil | 580 chronic epileptics, residing in the Curitiba area, previously evaluated by the author. | 210 epileptic patients randomly selected from the source population. | 14-82 years | International System of Classification 6 | Clinical history and examination, and EEG. | CT scan. | CT scan positive for NCC-compatible lesions. |
| Gulati *et al*  [74] | 1991 | Cohort | India | 517 patients with secondary epilepsy referred to the NMR Research Centre in Lucknow, for MRI. | 170 paediatric patients diagnosed with secondary epilepsy. | 6-12 years | Unspecified | Clinical history and neurological examination. | CT scan and MRI. | CT scan or MRI positive for NCC-compatible lesions. |
